# Facile Preparation of TiO_2_NTs/Au@MOF Nanocomposites for High-Sensitivity SERS Sensing of Gaseous VOC

**DOI:** 10.3390/s24144447

**Published:** 2024-07-10

**Authors:** Chunyan Wang, Yina Jiang, Yuyu Peng, Jia Huo, Ban Zhang

**Affiliations:** 1 Chongqing University Cancer Hospital, Chongqing 400044, China; 2 College of Pharmacy and Bioengineering, Chongqing University of Technology, Chongqing 400054, China; 3 Chongqing DeWen ZhiShang Education Technology Co., Ltd., Chongqing 400042, China; 4 College of Economics, Mongolian University of Life Sciences, Ulaanbaatar 17024, Mongolia

**Keywords:** surface-enhanced Raman spectroscopy (SERS), gas, volatile organic compounds, nanocomposite

## Abstract

Surface-enhanced Raman spectroscopy (SERS) is a promising and highly sensitive molecular fingerprint detection technology. However, the development of SERS nanocomposites that are label-free, highly sensitive, selective, stable, and reusable for gaseous volatile organic compounds (VOCs) detection remains a challenge. Here, we report a novel TiO_2_NTs/AuNPs@ZIF−8 nanocomposite for the ultrasensitive SERS detection of VOCs. The three-dimensional TiO_2_ nanotube structure with a large specific surface area provides abundant sites for the loading of Au NPs, which possess excellent local surface plasmon resonance (LSPR) effects, further leading to the formation of a large number of SERS active hotspots. The externally wrapped porous MOF structure adsorbs more gaseous VOC molecules onto the noble metal surface. Under the synergistic mechanism of physical and chemical enhancement, a better SERS enhancement effect can be achieved. By optimizing experimental conditions, the SERS detection limit for acetophenone, a common exhaled VOC, is as low as 10^−11^ M. And the relative standard deviation of SERS signal intensity from different points on the same nanocomposite surface is 4.7%. The acetophenone gas achieves a 1 min response and the signal reaches stability in 4 min. Under UV irradiation, the surface-adsorbed acetophenone can be completely degraded within 40 min. The experimental results demonstrate that this nanocomposite has good detection sensitivity, repeatability, selectivity, response speed, and reusability, making it a promising sensor for gaseous VOCs.

## 1. Introduction

In the current field of public health and health monitoring, the detection of volatile organic compounds (VOCs) has become an important research direction [1,2]. VOCs in exhaled breath, as a kind of biomarker, can reflect the physiological and pathological states inside the human body through their concentration and composition [3]. Therefore, accurate detection of gaseous VOCs is of great significance in early disease diagnosis, health risk assessment, as well as monitoring of treatment effects [4].

The performance comparison of multiple traditional VOC detection technologies is shown in Table 1. Even though gas chromatography/mass spectrometric techniques are the gold standard for VOC biomarker identification, these systems are complex and expensive and therefore have not emerged for routine clinical use. These devices use electrochemistry [5] or infrared (IR) spectroscopy [6] to detect simple single volatile compounds, and while these systems have reduced instrumental complexity, they cannot analyze complex VOC mixtures at lower abundance. Electronic noses (eNoses) and colorimetric sensor arrays can analyze VOC mixtures at low abundance using multiple relatively non-specific chemical sensors [7,8]. However, eNoses measure an aggregate response to VOC mixtures, and not the mixture composition. Furthermore, eNose sensor arrays typically require elaborate calibration and cannot be readily adapted to new or revised VOC signatures. Therefore, the development of a rapid, sensitive, and user-friendly method for detecting gaseous VOCs has become an urgent need in the field of scientific research. Surface-enhanced Raman scattering (SERS) technology, characterized by its speed, simplicity, high sensitivity, and molecular fingerprinting, has shown tremendous potential for applications in the field of biochemical detection [9,10]. Through the plasmon resonance effect on the surface of nanometal, SERS technology can greatly enhance the Raman scattering signal of the target molecule, enabling the detection of trace amounts of substances. Therefore, the detection efficiency of SERS technology highly depends on the performance of the substrate sensor material. However, due to the rapid movement rate of gases, it is difficult for the traditional noble metal SERS sensor to fully adsorb the gases, resulting in low detection sensitivity. To address this issue, researchers have conducted numerous studies in recent years. Zhang Zhen [11] proposed a SERS sensor based on dendritic silver nanocrystals, utilizing their highly specific surface area and excellent SERS activity to achieve the detection of 0.1 ppb acetaldehyde. Wang Tie [12] introduced a SERS sensor based on the metal-organic framework MIL−100(Fe), which uses its specific adsorption and Raman enhancement effect on methanethiol to realize the detection of 0.5 ppb methanethiol. Qiao Xuezhi [13] proposed a SERS sensor based on a hollow Co-Ni layered double hydroxide (LDH) nanocage on Ag nanowires (Ag@LDH), using its high-density hotspots and stable surface chemical modification to quickly detect 1.9 ppb aldehyde. Snikta [14] developed a hybrid SERS platform of Si membrane and Ag NPs with a high density of ‘hot spots’ for molecular trace detection 0.5 ppb anisole vapors. In recent years, SERS technology has achieved some results in the detection of VOCs in exhaled breath [15,16,17,18]. In recent years, research on substrate sensor material suitable for gaseous VOC SERS detection has mainly focused on irregular-shaped noble metals, utilizing their irregular morphology to slow down the movement rate of gases on the surface and further enhance detection sensitivity [19,20,21,22,23,24]. However, current SERS sensor material for gaseous VOC detection still suffer from issues such as insufficient sensitivity, poor selectivity, complex preparation, and non-reusability. Therefore, exploring novel and efficient SERS sensor materials is crucial for achieving high-sensitivity detection of gaseous VOCs.

In recent years, nanocomposites have made significant progress in the field of SERS sensing. Among them, TiO_2_ nanotubes (TiO_2_NTs) have attracted attention due to their unique photoelectric properties, highly specific surface area, and good chemical stability. Metal-organic frameworks (MOF) materials have demonstrated advantages in gas adsorption and separation due to their high specific surface area, porous structure, and tunability [25]. Additionally, gold nanoparticles (Au NPs) are traditional SERS-active substrates, and their enhancement effects have been widely recognized. Given this, this paper presents a novel TiO_2_NTs/Au@MOF nanocomposite as a SERS sensing structure for gaseous VOCs. The three-dimensional TiO_2_ nanotube structure with a large specific surface area provides abundant sites for the loading of Au NPs, which possess excellent local surface plasmon resonance (LSPR) effects, further leading to the formation of a large number of SERS active hotspots. The externally wrapped porous MOF structure adsorbs more gaseous VOC molecules onto the precious metal surface. Under the synergistic mechanism of physical and chemical enhancement, a better SERS enhancement effect can be achieved. The development of efficient and controllable synthesis methods for this material has been undertaken, and its potential for high-sensitivity sensing applications in the field of gaseous VOC detection is explored. It is expected that these nanocomposites can be applied to a wider range of fields, making greater contributions to early disease warning, health monitoring, and environmental monitoring.

**Table 1:** Comparison of common VOC detection methods.

Detection Method	Sensor	Test Sample	Detection Limit	Cost	Specificity	Complexity
GC		VOC [26]	200 ppm	Higher	Excellent	Higher
Absorption Spectrophotometry	BTX	Toluene [27]	8.1 ppm	Higher	Poor	Higher
Metal oxide	Co-doped In_2_O_3_ NFs	Ethanol [28]	100 ppm	Lower	Excellent	Lower
Photoacoustic	MOF@LiNb O_3_SAW	CO_2_, CH_4_ [29]	1.44 ppm, 0.08 ppm	Higher	Poor	Higher
Electrochemical	Co_9_S_8_/In_2_S_3_	Triethylamine [30]	20 ppm	Lower	Poor	Lower

## 2. Materials and Methods

### 2.1. Materials and Apparatus

Deionized water (DI) was used for all the aqueous solutions. Titanium foil (99.9%) was purchased from Beijing Xinruige Material Co., Ltd., Beijing, China, the calibrated standard acetophenone gas was purchased from Chengdu Taiyu Industrial Gas Co., Ltd., Chengdu, China, hydrogen tetrachloroauric acid (HAuCl_4_·4H_2_O, AR) was purchased from Sinopharm Chemical Reagent Co., Ltd., Shanghai, China, poly dimethyl diallyl ammonium chloride (PDDA, wt 35%, AR) was purchased from Heowns Biochemical Technology Co., Ltd. and all the other reagents were of analytical regent (AR) grade and purchased from Chongqing Chuandong Chemical Group, Chongqing, China. We also used a muffle furnace (SGM·M6/10, Sigma High-Temperature Electric Furnace Co., Ltd., Shanghai, China), DC power supply (2290-5, Tektronix, Beaverton, OR, USA), Ultrasonic cleaner (YQ-020A, Shanghai Yi Jing ultrasonic Instrument Co., Ltd., Shanghai, China), Raman spectrometer (LabRAM HR, HORIBA Jobin Yvon, Palaiseau, France), and scanning electron microscope (GeminiSEM 500, ZEISS, Oberkochen, Germany).

### 2.2. Preparation of TiO_2_NTs

An amount of 250 mL of deionized water and 250 mL of glycerol were poured into a conical flask, and 5 g of ammonium fluoride was added to prepare a 500 mL electrolyte solution. Small square titanium sheets (0.8 cm × 0.8 cm) were cut and ultrasonically cleaned in acetophenone, anhydrous ethanol, and deionized water for 10 min, respectively, and then dried. Subsequently, using the anodization method, the titanium sheet was connected to the positive pole of the DC power supply, and the platinum electrode was connected to the negative pole of the DC power supply. Electrolysis was carried out at a direct current voltage of 30 V for 1 h. After electrolysis, the titanium sheets were rinsed with deionized water and dried before being placed in a muffle furnace. They were annealed in air at 450 °C for 2 h, then taken out and ultrasonically cleaned in anhydrous ethanol for 10 s [31].

### 2.3. Preparation of Gold Nanoparticles (AuNPs)

An amount of 100 mL of 0.01% chloroauric acid solution was placed on a magnetic stirrer and stirred continuously until boiling. Then, 1 mL of 1% trisodium citrate solution was quickly added, and stirring was continued for 15 min. Stirring was stopped and the solution was cooled to room temperature before being stored in a refrigerator at 4 °C [32].

### 2.4. Preparation of TiO_2_NTs/AuNPs

The TiO_2_NTs were immersed in a 2% PDDA solution for 30 min and then washed with deionized water. Subsequently, the nano-gold suspension was poured onto the surface of TiO_2_NTs modified with PDDA and left to stand for 2 h. After that, the TiO_2_NTs were washed several times with deionized water and stored for future use [33].

### 2.5. Preparation of TiO_2_NTs/AuNPs@ZIF−8

A total of 0.9852 g of 2-methylimidazole and 0.4461 g of zinc nitrate hexahydrate were added to 30 mL of methanol solution. After thorough mixing, the resulting reaction solution was obtained [34]. Then, three TIO_2_NTS/Au substrates prepared under the same conditions were simultaneously put into the reaction solution. After reacting for 15 min, 30 min, and 45 min at 25 °C, the corresponding three TIO_2_NTS/Au@ZIF−8 substrates were obtained.

### 2.6. Characterization and Testing of TiO_2_NTs/AuNPs@ZIF−8 Nanocomposites

The prepared TiO_2_NTs/AuNPs@ZIF−8 nanocomposites were characterized using scanning electron microscopy (SEM), and further composition characterization of the prepared composite sensor was conducted using energy-dispersive X-ray spectroscopy (EDS). Then, acetophenone was used as a test sample, and the sensitivity, reproducibility, response speed, and photocatalytic degradation experiments of the prepared TiO_2_NTs/AuNPs@ZIF−8 sensor were conducted using confocal Raman spectroscopy. During the SERS test of gaseous acetophenone, the commercialized gaseous acetophenone that was precisely calibrated was used as the test sample. The SERS sensing substrate was placed in advance in a small closed box with two openings, one for the inlet and the other for the outlet. The inlet was connected to the gaseous acetophenone, while the outlet was connected to an air bag to collect exhaust gas. All Raman tests used an excitation wavelength of 532 nm, a power of 10 mw, an integration time of 1 s, and a cumulative count of 1.

## 3. Results and Discussion

### 3.1. Synthesis and Characterizations

For the preparation of the TiO_2_/Au@ZIF−8 nanocomposite, TiO_2_NTs structures were mass-produced with anodic oxidation, and a sufficient amount of gold nanoparticle was prepared using a traditional chemical reduction method. This preparation allows for multiple uses. Subsequently, the self-assembly technique was employed, as specified below: the surface of TiO_2_NTs are modified with a cationic polymer, PDDA, and subsequently, and negatively charged gold nanoparticles were adsorbed onto the upper surface and inner surface of the nanotubes, achieving three-dimensional distribution of gold nanoparticles. To further adsorb gaseous VOCs, an in-situ deposition method was used to coat the gold particles with a layer of ZIF−8 shell. During the synthesis process, the pore size of the TiO_2_ NTs is related to the applied external voltage, the particle size of the gold nanoparticles depends on the volume of the reducing agent, and the thickness of the coated ZIF−8 shell depends on the deposition time. The entire preparation process is controllable and structured, requiring no expensive equipment, and can be completed in a regular laboratory. Additionally, it allows for batch production with simple and convenient operation.

**Figure 1:**
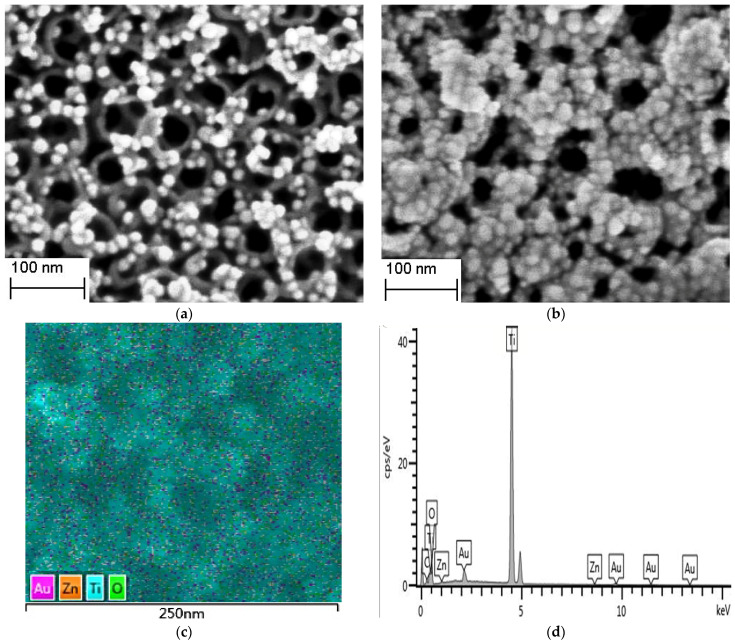
SEM images of TiO_2_NTs/AuNPs (**a**) and TiO_2_NTs/AuNPs@ZIF−8 nanocomposites obtained at 30 min (**b**); EDS element distribution map (**c**) and Spectrum (**d**) of TiO_2_NTs/AuNPs@ZIF−8.

To observe the morphology and size of the prepared composite structures, SEM images of TiO_2_NTs/AuNPs and TiO_2_NTs/AuNPs@ZIF−8 are shown in Figure 1a,b, respectively. In Figure 1a, it is evident that the dispersed nanoparticles are uniformly distributed on the upper surface and inner walls of the regularly aligned nanotubes. By employing particle size distribution software, the nanotube diameters and the diameters of the gold nanoparticles on the surface were measured, revealing an average nanotube diameter of 50 nm and an average gold nanoparticle size of 15 nm. Upon coating with the ZIF−8 shell, the nanoparticles in Figure 1b exhibit bright cores surrounded by lighter-colored shells. Applying the particle size distribution analysis software, the particle sizes of each nanoparticle with the shell were statistically analyzed, and the average value was calculated. By subtracting the average particle size of the gold core from this average value and dividing the difference by 2, the average ZIF−8 *shell thickness was determined to be 6 nm. To validate the element distribution of the composite structure, an EDS analysis was performed on the TiO_2_NTs/AuNPs@ZIF−8 nanocomposite, as shown in* Figure 1c. *The image demonstrates a uniform distribution* of elements including Zn, Au, Ti, C, and O across the composite surface. Furthermore, the EDS spectrum in Figure 1d provides a clear overview of the composition and corresponding content of these elements in the nanocomposite, confirming the successful encapsulation of ZIF−8 on the TiO_2_NTs/Au surface. In summary, the obtained composite structure comprises TiO_2_NTs/AuNPs@ZIF−8.

### 3.2. Regulation of the Thickness of ZIF−8 Shell in the Nanocomposites

**Figure 2:**
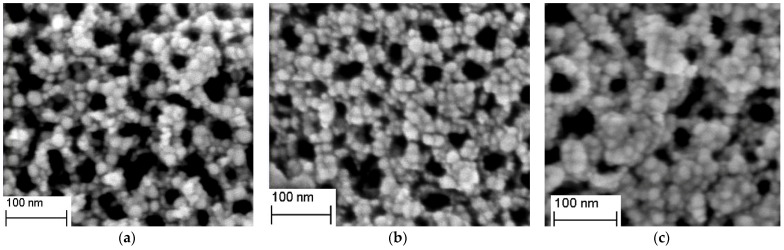
SEM image of TiO_2_NTs/AuNPs@ZIF−8 nanocomposites obtained at three different deposition times: 15 min (**a**), 30 min (**b**), and 45 min (**c**), respectively.

**Figure 3:**
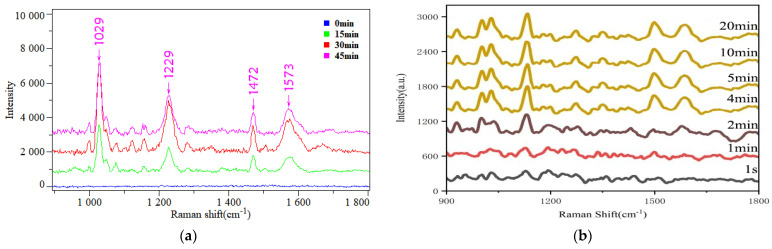
(**a**) The SERS spectra of 1 ppm gaseous acetophenone adsorbed on the surface of TiO_2_NTs/AuNPs@ZIF−8 nanocomposites with different ZIF−8 shell thicknesses for 30 min; (**b**) SERS spectra of 1 ppb gaseous acetophenone adsorbed on TiO_2_NTs/AuNPs@ZIF−8 nanocomposites with ZIF−8 shell thickness of 6 nm for different times.

In the composite nanostructure designed in this study, the thickness of the ZIF−8 shell plays a crucial role in determining the number of adsorbed gaseous VOC molecules, ultimately affecting the performance of SERS detection. To quantitatively assess this effect, we first manipulated the thickness of ZIF−8 shells by precisely controlling the deposition time of ZIF−8. Subsequently, scanning electron microscopy (SEM) was employed to characterize the morphology and particle size of the nanostructures (Figure 2). The ZIF−8 shell thickness was further calculated using a method consistent with Section 3.1. The results indicate that, for deposition times of 15 min, 30 min, and 45 min, the shell thicknesses are approximately 3.5 nm, 6 nm, and 9.4 nm, respectively (Figure 2a–c). Notably, the thickness of the ZIF−8 shell increases progressively with the extension of deposition time. Specifically, when the deposition time reaches 45 min, the nanoparticles exhibit a near-complete interconnected state, a phenomenon attributed to the thickening of the outer shell. To further investigate the impact of different ZIF−8 shell thicknesses on the SERS detection performance of gaseous VOCs, this article employed nanocomposites with varying ZIF−8 shell thicknesses as SERS enhancement substrates under identical experimental conditions. As a test sample, 1 ppm of gaseous acetophenone was adsorbed onto the surfaces of TiO_2_NTS/Au@ZIF−8 substrates with varying shell thicknesses for 30 min each. Following adsorption, SERS measurements were promptly conducted under identical testing conditions, and the results are presented in Figure 3a. It was found that, during a 30-min adsorption period, nanocomposites devoid of a ZIF−8 shell exhibit negligible Raman enhancement for acetophenone. However, as the thickness of the ZIF−8 shell increases, the SERS enhancement effect exhibited by the nanocomposites towards acetophenone gradually improves. The Raman peaks at 1029 cm^−1^, 1229 cm^−1^, 1423 cm^−1^, 1470 cm^−1^, 1573 cm^−1^, and 1669 cm^−1^ correspond to the out-of-plane bending vibration of C-H in the benzene ring, the stretching vibration of C-C in the benzene ring, the in-plane bend vibration of C-C-H and the deformation vibration of CH_3_, another bend vibration of C-C-H, the stretching vibration of Ring C-C, and the stretching vibration of the ketone group (C=O), respectively [35]. Under the four conditions, the SERS signal intensity of gaseous acetophenone molecules collected at a MOF shell thickness of 6 nm was larger. As the thickness of the ZIF−8 shell increased further, a decreasing trend was observed in the SERS enhancement effect towards acetophenone, indicating that the ZIF−8 shell in the nanocomposites directly impacts the detection efficiency of gaseous VOCs. This phenomenon can be attributed to the porous structure of ZIF−8, which boasts a highly specific surface area and the ability to restrict gas flow on the surface of noble metals, thereby enhancing the adsorption of gaseous VOCs onto the noble metal surface. Specifically, when the ZIF−8 film is thin, fewer gas molecules are adsorbed on the outer surface of the gold nanoparticles, leading to inadequate chemical enhancement. Conversely, as the thickness of the ZIF−8 film increases, more gas molecules can be confined within the porous structure. However, an excessively thick ZIF−8 shell layer may hinder the direct contact between VOC molecules and the surface of Au nanoparticles [36,37]. This implies that VOC molecules encounter a longer diffusion path through thicker ZIF−8 shell layers, diminishing their likelihood of adsorption onto the Au nanoparticle surface and subsequently reducing the number of molecules participating in SERS scattering. Furthermore, the thicker ZIF−8 shell layer may exacerbate the attenuation of scattered light as it traverses the shell. As the scattered light propagates through the ZIF−8 shell, its intensity diminishes due to absorption, scattering, and other material-specific processes, leading to a decrease in the number of photons detected, ultimately resulting in a weaker SERS signal intensity.

### 3.3. The Impact of Adsorption Time on the Effectiveness of SERS Testing for Gaseous Acetophenone

The number of gas molecules adsorbed on the surface of the nanocomposites is positively correlated with its SERS signal intensity. The amount of adsorbed gas molecules is not only related to the performance of the substrate itself but also directly related to the adsorption time. To assess the kinetics of gaseous acetophenone adsorption onto the fabricated nanocomposites, this study prepared nanocomposites under standardized conditions and exposed them to 1 ppb of gaseous acetophenone for varying time durations. To enhance the detection stability and reliability, twenty distinct points were selected on each nanocomposite surface for SERS testing under identical testing conditions. The resulting SERS signal for a given duration was obtained by averaging the twenty signals and applying filtering techniques, representing the measured signal after 1 ppb of gaseous acetophenone adsorbed onto the nanocomposite surface for that particular time. The data presented in Figure 3b demonstrate a definitive trend in the relationship between adsorption time and the signal intensity of acetophenone. As the duration of adsorption increases, it is evident that the intensity of the acetophenone SERS signal gradually intensifies. Specifically, once the adsorption time reaches 4 min, the signal intensity remains largely stable, suggesting a saturation point. This can be attributed to the porous nature of ZIF−8, which offers a significant number of adsorption sites for acetophenone molecules. In the initial stages of the adsorption process, when gaseous acetophenone molecules encounter the TiO_2_NTs/Au@ZIF−8 nanocomposite, they begin to adsorb onto the surface. However, due to the small number of adsorbed gaseous acetophenone molecules, the obtained acetophenone SERS signal is relatively weak. As the adsorption time progresses, more and more gaseous acetophenone molecules are adsorbed onto the surface of the nanocomposite. Since the intensity of the SERS signal is directly influenced by the number of molecules participating in the scattering process, the measured signal intensity also increases accordingly. When the adsorption sites reach saturation, it becomes difficult for newly added acetophenone molecules to find more adsorption sites, and the adsorption rate and desorption rate reach a dynamic equilibrium. Under this equilibrium state, the population of acetophenone molecules within the enhancement region of the SERS nanocomplex undergoes dynamic minor variations. Nevertheless, these minor fluctuations are typically insufficient to have a significant impact on the intensity of the SERS signal. However, it is important to note that such minute fluctuations may still have a slight impact on the stability of the SERS signal. To address this issue and enhance the stability and reliability of the SERS signal, this paper adopts strategies such as conducting multiple measurements and averaging the results, as well as applying spectral filtering techniques to the collected data. These methods effectively minimize the influence of random errors and ensure a more stable and reliable SERS signal.

### 3.4. Evaluation of the SERS Performance of Nanocomposites for Gaseous VOCs

**Figure 4:**
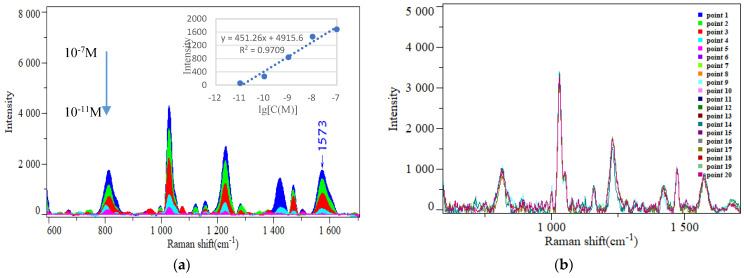

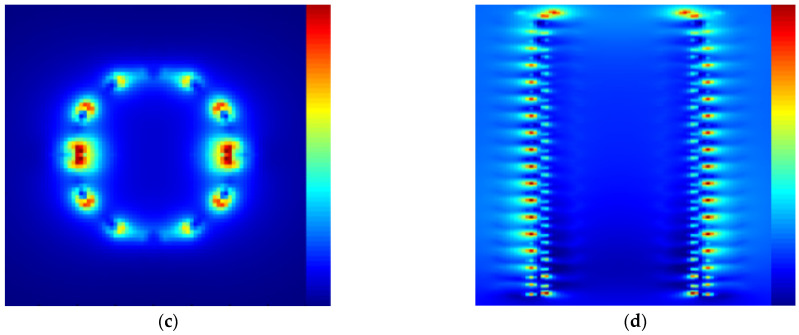
(**a**) SERS spectra of different concentrations of acetophenone on the surface of the TiO_2_NTs/AuNPs@ZIF−8 nanocomposites. Inner picture is the plot of the intensity of SERS peak at 1025 cm^−1^ versus the logarithm of acetone concentration. (**b**) SERES spectra measured at 20 different spots on the surface of the TiO_2_NTs/AuNPs@ZIF−8 nanocomposites adsorbed with acetophenone (color insert shows Raman mapping of the substrate surface at 20 different points). The electromagnetic simulation diagrams of the TiO_2_NTs/AuNPs@ZIF−8 composite nanostructure: (**c**) top view; (**d**) front view.

Sensitivity, reproducibility, selectivity, and reusability serve as fundamental parameters for evaluating the performance of nanocomposites as SERS substrates for gaseous VOCs. To further observe the relationship between the Raman signal intensity of acetophenone and its concentration, different concentrations of acetophenone were used as test samples, and TiO_2_NTs/AuNPs@ZIF−8 (with ZIF−8 shell thickness of 6 nm) was employed as the SERS sensor. Under identical conditions, SERS testing was conducted, and the results are shown in Figure 4a. It could be found that the prepared TiO_2_NTs/AuNPs@ZIF−8 have a significant enhancement effect on the Raman signal of acetophenone. All samples displayed distinct Raman peaks characteristic of acetophenone, exhibiting identical peak positions. Notably, the Raman signal intensity was observed to decrease with decreasing acetophenone concentration, indicating a strong linear correlation (R^2^ = 0.9709, using the 1573 cm^−1^ Raman peak as the reference). When the concentration is reduced to 10^−11^ M, a weak Raman peak of acetophenone can be observed, but when it is further diluted to 10^−12^ M, almost no Raman peak of acetophenone can be observed. Therefore, it is concluded that the TiO_2_NTs/AuNPs@ZIF−8 have a detection limit of gaseous acetophenone as low as 10^−11^ M. This detection limit is lower than the content of acetophenone in the exhaled breath of breast cancer patients, indicating that the prepared nanocomposites can be used for direct detection of acetophenone in the exhaled breath. Based on the SERS enhancement mechanism, the SERS enhancement effect of this SERS nanocomposite originates from electromagnetic enhancement and chemical enhancement, with electromagnetic enhancement playing a dominant role. In order to explore the SERS electromagnetic enhancement mechanism of the as-prepared SERS nanocomposites, finite-different time-domain (FDTD) was used to analyze the electromagnetic field enhancement. The boundary condition was set as the perfectly matched layer. The incident light was set as a plane wave with a 532 nm wavelength, with propagation in the x-direction and polarization in the z-direction. The spatial parameters were set as follows: the time step was 0.0006 fs; the mesh size was 1 nm; the background index was 1.0 (air). Figure 4c,d shows the great enhancement of the electromagnetic field between two Au NPs on the surface of TiO_2_NTs, which is called the “hot spot”. Due to the dense and uniform distribution of Au NPs on the surface of TiO_2_NTs, a large number of “hot spots” are formed in the three dimensional space, thus improving the sensitivity of the substrate.

Detection reproducibility is another important evaluation index for detection effect of the SERS sensor. The TiO_2_NTs/AuNPs@ZIF−8 SERS nanocomposite was selected, and surface adsorption of acetophenone was carried out. Twenty different positions on the nanocomposite surface were selected for SERS testing, and the results are shown in Figure 4b. It can be found that all SERS spectra corresponding to acetophenone have the same Raman peak and their intensities are basically consistent with a RSD of 4.5%. This indicates that the TiO_2_NTs/AuNPs@ZIF−8 SERS sensor has good uniformity and detection reproducibility.

**Figure 5:**
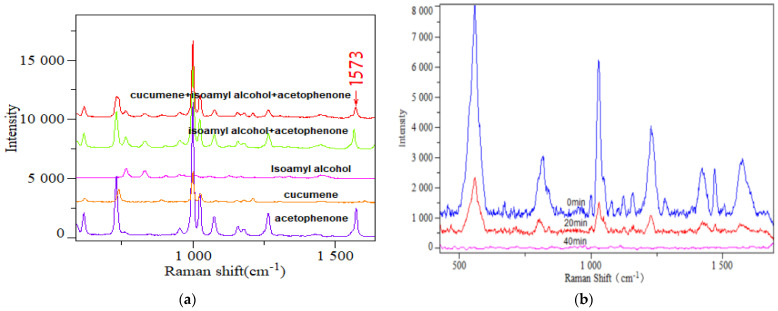
(**a**) The SERS spectra of three common exhaled VOCs and their mixtures, adsorbed on the surface of the nanocomposites, respectively. (**b**) SERS spectra of 10^−7^ M acetophenone on the TiO_2_NTs/AuNPs@ZIF−8 surface under UV light irradiation at different times.

To further evaluate the detection selectivity of gaseous VOCs on the surface of the SERS nanocomposites, this article used the nanocomposites prepared under the same conditions as the enhancement substrate. Three commonly exhaled VOCs and their mixtures were used as test samples separately. Under the same adsorption and testing conditions, the measured SERS spectra are shown in Figure 5a. It could be observed that they all exhibited corresponding Raman peaks. Among them, Raman peaks appeared at 1573 cm^−1^ in the SERS spectra of acetophenone, the mixture of acetophenone and isoamyl alcohol, and the mixture of all three VOCs, but this peak did not appear in the SERS spectra of the other two VOCs alone. This indicates that the Raman peak at 1573 cm^−1^ can be used as a characteristic peak for acetophenone, and the substrate prepared in this study has a certain selectivity for the detection of acetophenone in VOC mixed gases containing acetophenone.

To verify the reusability properties of the composite sensor, this study placed the TiO_2_NTs/AuNPs@ZIF−8 sensor that adsorbed 10 min of gaseous acetophenone (10 ppm) under UV light irradiation, and performed SERS detection at different irradiation times. The results are shown in Figure 5b. It could be found that as the irradiation time increases, the SERS signal intensity of acetophenone on the nanocomposite surface gradually decreases. When the irradiation reaches 40 min, the Raman signal of acetophenone is no longer visible. This is because acetophenone is an organic compound, and TiO_2_ has good photocatalytic degradation efficiency. Under UV light irradiation, the adsorbed organic compounds on the surface can be photocatalytically degraded into CO_2_ and water [38], so the signal of acetophenone finally disappears. This demonstrated that the TiO_2_NTs/AuNPs@ZIF−8 nanocomposite, having adsorbed VOCs, exhibited exceptional capacity to degrade the adsorbed VOCs from its surface after 40 min of exposure to ultraviolet light. Furthermore, this degradation process enabled the nanocomposite to be reused multiple times, thereby significantly reducing costs and enhancing its overall cost-effectiveness.

## 4. Conclusions

Volatile organic compounds (VOCs), as crucial biomarkers for exhaled breath diseases, hold significant research value in their high_sensitivity, high_selectivity, and reproducible SERS testing. The TiO_2_NTs/Au@ZIF−8 nanocomposite has a detection limit for acetophenone down to 10^−11^ M, which is lower than the content of acetophenone in the breath of healthy individuals, and a good linear relationship was observed between the measured SERS signal intensity of acetophenone and its concentration. The same nanocomposite showed good SERS reproducibility for acetophenone detection, with a relative standard deviation (RSD) of 4.5% among 20 different test points, indicating that the nanocomposite has good repeatability. Moreover, the nanocomposite responded to gaseous acetophenone within 1 min, and the response signal stabilized after 4 min; additionally, UV irradiation for 40 min successfully desorbed the acetophenone molecules on the surface of the sensor film, allowing for repeated use. The results show that the composite nano-sensing structure proposed in this paper has good performance in terms of sensitivity, homogeneity, response speed, and reusability for gaseous VOC detection, which provides good technical support for the application of SERS technology in the field of breath VOC detection. Based on this current study, future efforts will focus on achieving efficient detection of target VOCs in complex exhaled breath samples for auxiliary diagnosis of diseases. To further enhance the specificity of detection, the pore structure of ZIF−8 in the nanocomposite can be further designed and tailored according to the types of target VOCs, aiming to precisely match their molecular dimensions. On this basis, it can further enhance the adsorption affinity through post-synthesis modification. Concurrently, by integrating the exceptional high- sensitivity fingerprint recognition capabilities of SERS detection technology, the efficient separation and qualitative and quantitative identification of target VOCs in exhaled breath is potentially achievable. This approach will lay a solid foundation for clinical applications in the auxiliary diagnosis of diseases.

## Data Availability

The data that support the findings of this study are available from the corresponding author upon reasonable request. The data are not publicly available due to privacy.
